# Utility of Multi-Parametric Quantitative Magnetic Resonance Imaging for Characterization and Radiotherapy Response Assessment in Soft-Tissue Sarcomas and Correlation With Histopathology

**DOI:** 10.3389/fonc.2019.00280

**Published:** 2019-04-25

**Authors:** Jessica M. Winfield, Aisha B. Miah, Dirk Strauss, Khin Thway, David J. Collins, Nandita M. deSouza, Martin O. Leach, Veronica A. Morgan, Sharon L. Giles, Eleanor Moskovic, Andrew Hayes, Myles Smith, Shane H. Zaidi, Daniel Henderson, Christina Messiou

**Affiliations:** ^1^Cancer Research UK Cancer Imaging Centre, Division of Radiotherapy and Imaging, The Institute of Cancer Research, London, United Kingdom; ^2^Department of Radiology, The Royal Marsden NHS Foundation Trust, Sutton, United Kingdom; ^3^Sarcoma Unit, Department of Radiotherapy and Physics, The Royal Marsden NHS Foundation Trust, London, United Kingdom; ^4^Division of Radiotherapy and Imaging, The Institute of Cancer Research, London, United Kingdom; ^5^Department of Surgery, The Royal Marsden NHS Foundation Trust, London, United Kingdom; ^6^Department of Histopathology, The Royal Marsden NHS Foundation Trust, London, United Kingdom

**Keywords:** neoplasm, soft tissue sarcoma, magnetic resonance imaging, radiation therapy, diffusion weighted MRI, apparent diffusion coefficient (ADC)

## Abstract

**Purpose:** To evaluate repeatability of quantitative multi-parametric MRI in retroperitoneal sarcomas, assess parameter changes with radiotherapy, and correlate pre-operative values with histopathological findings in the surgical specimens.

**Materials and Methods:** Thirty patients with retroperitoneal sarcoma were imaged at baseline, of whom 27 also underwent a second baseline examination for repeatability assessment. 14/30 patients were treated with pre-operative radiotherapy and were imaged again after completing radiotherapy (50.4 Gy in 28 daily fractions, over 5.5 weeks). The following parameter estimates were assessed in the whole tumor volume at baseline and following radiotherapy: apparent diffusion coefficient (ADC), parameters of the intra-voxel incoherent motion model of diffusion-weighted MRI (D, *f*, D^*^), transverse relaxation rate, fat fraction, and enhancing fraction after gadolinium-based contrast injection. Correlation was evaluated between pre-operative quantitative parameters and histopathological assessments of cellularity and fat fraction in post-surgical specimens (ClinicalTrials.gov, registration number NCT01902667).

**Results:** Upper and lower 95% limits of agreement were 7.1 and −6.6%, respectively for median ADC at baseline. Median ADC increased significantly post-radiotherapy. Pre-operative ADC and D were negatively correlated with cellularity (*r* = −0.42, *p* = 0.01, 95% confidence interval (CI) −0.22 to −0.59 for ADC; *r* = −0.45, *p* = 0.005, 95% CI −0.25 to −0.62 for D), and fat fraction from Dixon MRI showed strong correlation with histopathological assessment of fat fraction (*r* = 0.79, *p* = 10^−7^, 95% CI 0.69–0.86).

**Conclusion:** Fat fraction on MRI corresponded to fat content on histology and therefore contributes to lesion characterization. Measurement repeatability was excellent for ADC; this parameter increased significantly post-radiotherapy even in disease categorized as stable by size criteria, and corresponded to cellularity on histology. ADC can be utilized for characterizing and assessing response in heterogeneous retroperitoneal sarcomas.

## Introduction

Soft-tissue sarcomas are often highly heterogeneous tumors with variable components that can include cellular tumor, fat, necrosis, and cystic change. In many soft-tissue sarcoma sub-types, post-treatment changes often cannot be described by standard size criteria (response evaluation criteria in solid tumors, RECIST 1.1), as components within responding tumors may not shrink, or may increase in size, after radiotherapy (1, 2). Additionally, where systemic therapies alone are administered in non-resectable disease (3) or where radiotherapy with systemic therapies are used as an alternative to surgery (4), sensitive and reliable non-invasive methods for response assessment are needed. Magnetic resonance imaging (MRI) enables non-invasive assessment of the whole tumor, and a multi-parametric approach can be used to quantify tumor components and assess changes within these components as tumors respond to treatment.

Diffusion-weighted MRI (DW-MRI) assessment of tumor cellularity and dynamic contrast-enhanced MRI (DCE-MRI) assessment of tumor vascularity have been shown to increase sensitivity of MRI in response assessment to neoadjuvant treatment in soft-tissue sarcomas (5). Contrast-enhancement has been shown to be indicative of response after isolated limb perfusion (6). The transverse relaxation rate, which is sensitive to paramagnetic deoxyhemoglobin and hypoxia (${\text{R}}_{2}^{*}$), has been shown to be predictive of radiotherapy response in pre-clinical studies (7). Recent recommendations have suggested quantitative MRI parameters as exploratory end-points in clinical trials but emphasized the requirement for further validation studies (8). There is a need, therefore, for assessment of the technical performance and clinical utility of quantitative MRI techniques in soft-tissue sarcomas in order to inform protocol development and selection of summary statistics for reporting. Optimization of quantitative imaging protocols requires knowledge of tumor properties, for example selection of diffusion-weightings (b-values) for estimation of apparent diffusion coefficients (ADCs) (9). Separate assessments of common sub-types are essential since optimal treatment may depend on histological sub-type (10). Importantly, quantitative MRI parameters require validation with histopathology to enable their future use in treatment planning and response assessment.

The aim of this study was to assess quantitative MRI techniques for characterization of retroperitoneal sarcomas by evaluating (i) quantitative MRI parameters in a typical mixed cohort and in the main sub-types; (ii) repeatability of parameters at baseline; (iii) post-radiotherapy changes in a cohort and individual tumors; (iv) the correlation between pre-operative quantitative imaging parameters and histopathology.

## Materials and Methods

### Patients

**Figure 1:**
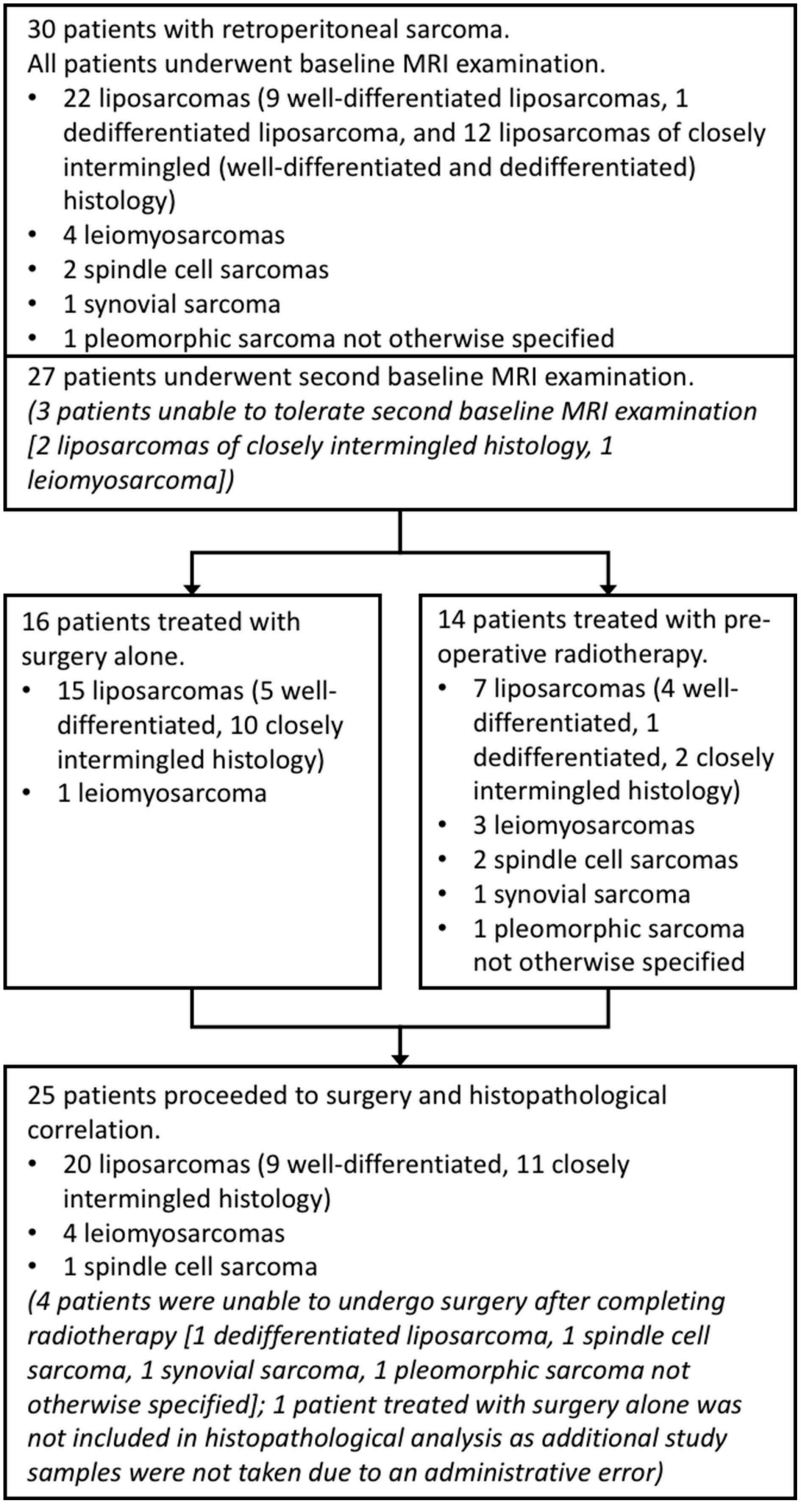
Study organization. Flow chart showing numbers of patients and tumor sub-types included in each part of the study. Well-differentiated liposarcomas refer to fatty neoplasms corresponding histologically with differentiated adipocytic tumors closely resembling mature fat, and dedifferentiated liposarcomas as more solid to myxoid tumors corresponding histologically with non-lipogenic, usually pleomorphic, tumors. In this study, closely intermingled tumors refer to intermingled well- and dedifferentiated components, which can be seen histologically.

Thirty patients with retroperitoneal sarcoma were included in this prospective single-center study. This study was carried out in accordance with the recommendations of the Royal Marsden Hospital committee for clinical research and approval from a national Research Ethics Committee (East of England—Cambridge East Research Ethics Committee). All subjects gave written informed consent in accordance with the Declaration of Helsinki (Clinical trials registry: ClinicalTrials.gov, registration number: NCT01902667). Sequential patients were identified between July 2013 and May 2016 by a multi-disciplinary team at a specialist sarcoma unit. Patients were eligible for inclusion if they had retroperitoneal sarcoma with planned surgical resection, with or without pre-operative radiotherapy. Exclusion criteria were contraindications for MRI or inability to tolerate the MRI examination. Two further patients were recruited but subsequently excluded as they did not meet the inclusion criteria (one was found not to have retroperitoneal sarcoma; one had a change in management). Patients underwent a baseline MRI examination, with a second baseline examination for repeatability assessment. Tumor types and numbers of patients are described in Figure 1. Patients treated with radiotherapy underwent another MRI examination after radiotherapy, prior to surgery (median interval between final radiotherapy fraction and MRI examination was 27 days, range 13–33 days). All MRI examinations took place between July 2013 and July 2016.

### Radiotherapy

Patients underwent a contrast-enhanced planning computed tomography (CT) scan to construct target volumes and organs at risk. Diagnostic MR images were co-registered to construct the gross tumor volume (GTV). The clinical target volume (CTV) included the GTV with a geometric expansion of 5 mm and adapted to encompass areas of microscopic disease. The planning target volume (PTV) included CTV plus an additional geometrical margin of 9 mm (anteriorly, medially) and 12 mm (superiorly, inferiorly, posteriorly, laterally) to take into account patient set-up errors and organ motion. A median dose of 50.4 Gy in 28 daily fractions, over 5.5 weeks was prescribed to the PTV using a 5-field intensity-modulated radiotherapy (IMRT) technique. Treatment verification was performed on days 1–3 and then weekly with on-board cone-beam CT imaging.

### Imaging

Patients were scanned on a 1.5 T MAGNETOM Aera MRI scanner (Siemens, Erlangen, Germany) equipped with a work-in-progress diffusion-weighted echo-planar imaging (DW-EPI) package. Patients were positioned supine using anterior body matrix and posterior spine matrix receiver coils. Following axial and coronal T_1_-weighted and T_2_-weighted anatomical imaging, quantitative imaging series were acquired: DW-MRI for ADC estimation; additional DW-MRI for estimation of intra-voxel incoherent motion (IVIM) parameters (diffusion coefficient D, volume fraction *f*, pseudo-diffusion coefficient D^*^); multiple gradient-echo imaging for ${\text{R}}_{2}^{*}$ estimation; Dixon imaging for fat fraction (FF) estimation; and pre- and post-contrast T_1_-weighted imaging for estimation of enhancing fraction (EF) and fractional enhancement (ε_F_) (11) (Supplementary Table 1). At baseline, DW-MRI and multiple gradient-echo imaging were repeated after a break during which the patient left the scanner room and was then repositioned. Acquisition time was 70 min for double-baseline examinations. One patient was contra-indicated for gadolinium-based contrast agents. For technical reasons, IVIM series could not be acquired in one patient at baseline, one patient post-radiotherapy, and one patient at baseline and post-radiotherapy. 10 patients did not have Dixon imaging as this was added to the imaging protocol during the study. Patients who did not undergo post-contrast, IVIM, or Dixon imaging were excluded from analysis of EF and ε_F_, IVIM, and FF, respectively, but included in other analysis.

### Whole-Tumor Image Analysis

Assessments of baseline values, repeatability, and post-radiotherapy changes were carried out using quantitative MRI parameters estimated from the whole tumor volume. Regions of interest (ROIs) were drawn on axial T_2_-weighted images by an experienced soft-tissue sarcoma radiologist (8). ROIs were drawn around the whole tumor on every slice on which the tumor appeared, then transferred to each imaging series, and combined to form a volume of interest (VOI). Tumor volumes were estimated from the total volume of voxels in the VOI. ADC and ${\text{R}}_{2}^{*}$ were estimated voxel-by-voxel using Levenberg-Marquardt least-squares mono-exponential fits. IVIM parameters were estimated voxel-by-voxel using a Markov-chain Monte Carlo method for robust bi-exponential curve-fitting. All ROI drawing and curve fitting was performed using proprietary software (Adept, Institute of Cancer Research, London, UK). Median, mean, standard deviation, 10th, 25th, 75th, and 90th centiles, skew and kurtosis of all fitted voxels in the VOI were reported. A signal intensity threshold was applied to exclude suppressed fat from ADC, IVIM, and ${\text{R}}_{2}^{*}$ as DW-MRI and multiple gradient-echo imaging employed fat suppression. Two tumors (well-differentiated liposarcomas) composed of more than 80% fat were excluded from ADC, IVIM, and ${\text{R}}_{2}^{*}$ analysis as they were not evaluable using fat-suppressed DW-MRI and multiple gradient-echo imaging. Signal-to-noise ratio (SNR) was estimated in the DW-MRI series used for ADC estimation. SNR was estimated in the lowest b-value images (*b* = 50 s mm^−2^) and was estimated as 0.66 × S_tumor_/SD_noise_ where S_tumor_ is the mean signal in the tumor ROI and SD_noise_ is the average of the standard deviation in two noise ROIs. Noise ROIs were drawn in the background near the corners of the image. SNR was averaged over all slices on which the tumor appeared, and averaged over all patients included in DW-MRI analysis. FF was calculated voxel-by-voxel as the ratio of signal in the Dixon fat image to the sum of signals in fat and water images and the mean taken across all voxels in the VOI. EF was defined as the fraction of voxels in the VOI increasing in signal intensity by more than 5% between pre- and post-contrast T_1_-weighted images. ε_F_ was defined as (S_1_ – S_0_)/(S_1_+S_0_), where S_0_ and S_1_ are the signal in a voxel in pre- and post-contrast images, respectively, as described in other studies (11). Tumor volume was estimated from the total volume of all voxels in the VOI.

### Statistics

All statistical analysis was carried out using Matlab 2016a, The MathWorks Inc., Natick, MA. Differences between the two main sub-types (liposarcomas and leiomyosarcomas) were assessed using Wilcoxon rank sum tests (ranksum, Matlab 2016a). Repeatability was assessed using the method of Bland and Altman (12). The coefficient of variation (CoV) of repeated baseline measurements $\text{CoV}=100\%\times \sqrt{exp\left(s_{W}^{2}\right)-1}$, and 95% limits of agreement $\text{LoA}=100\%\times \left[exp\left(\pm 1.96\sqrt{2}s_{W}\right)-1\right]$, were used to quantify repeatability, where *s*_*W*_ is the within-subject standard deviation $s_{W}=\sqrt{\frac{1}{2N}\overset{N}{\underset{i=1}{\sum }}d_{i}^{2}}$, with *d*_i_ the difference between two baseline measurements for the *i*th patient, and *N* the number of patients (13). Repeatability of median, mean, standard deviation, and 10th to 90th centiles was assessed using the natural logarithm of the values and reported on a percentage scale. 95% LoA of skew and kurtosis were estimated using untransformed values and reported as absolute changes. 95% confidence intervals were estimated for CoV and LoA (14).

Radiotherapy response was assessed clinically using RECIST 1.1 criteria using T_2_-weighted images on a picture archiving and communication system (PACS) workstation (15). Post-radiotherapy changes in quantitative MRI parameters in the cohort were assessed using Wilcoxon signed rank tests (signrank, Matlab 2016a). *p* < 0.05 was used to indicate significance. Post-radiotherapy changes in individual patients were identified by comparison with the 95% LoA of repeated baseline measurements.

### Histopathological Analysis and Imaging Correlation

**Figure 2:**
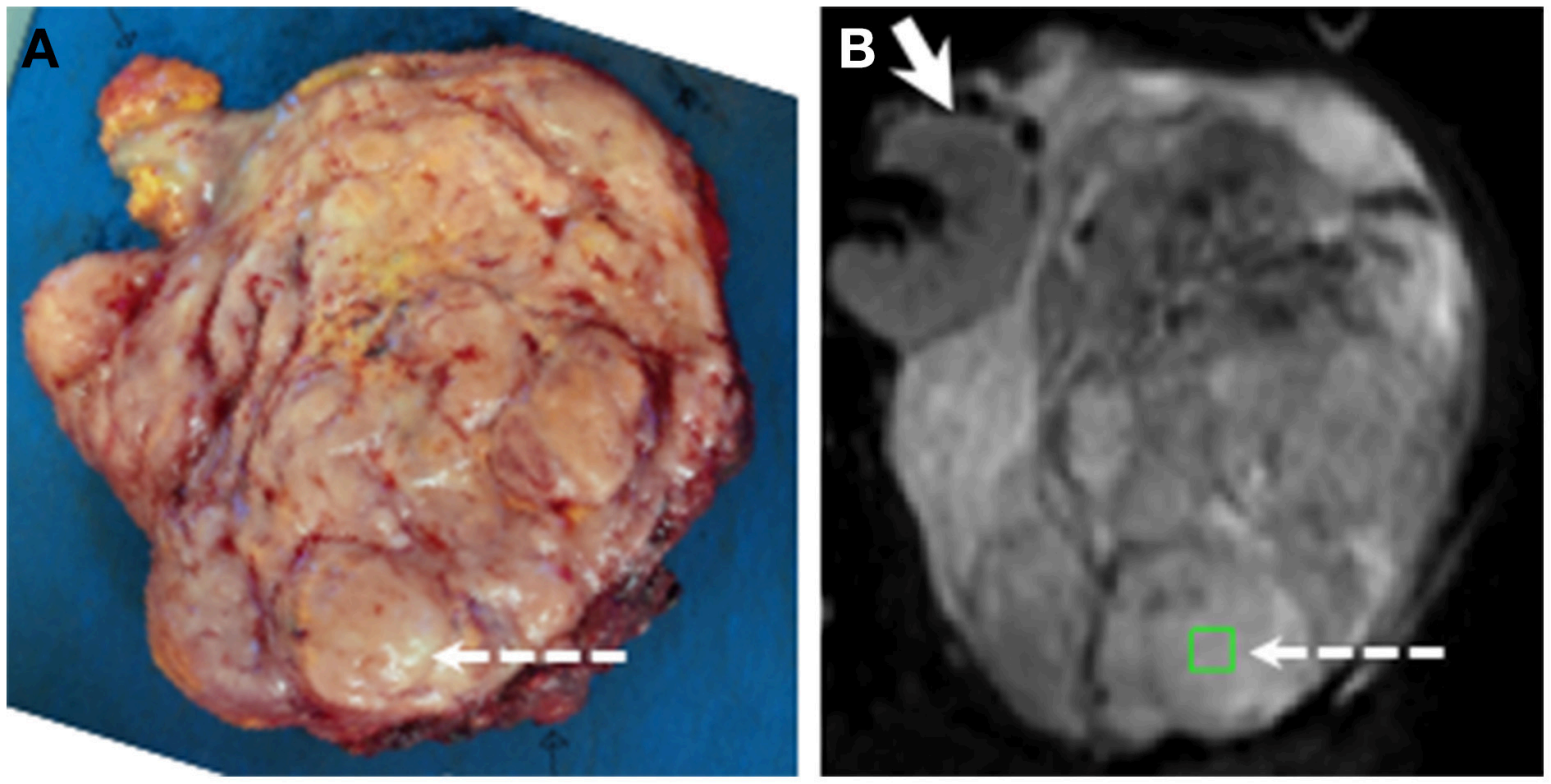
Example showing positioning of ROI for histopathological and radiological analysis. **(A)** Slice cut by histopathologist. **(B)** fitted *b* = 0 s mm^−2^ image from matching slice in DW-MRI series. ROI shown by green square and dashed arrows. Solid arrow in **(B)** shows kidney (not removed in surgery). ROIs were chosen jointly by the histopathologist and radiologist using pre-surgical imaging, markers inserted by the surgeon, and anatomical landmarks within tumor. Note ROI lies in a nodule in the posterior part of the tumor.

A representative axial slice of the tumor was selected by an experienced soft-tissue sarcoma radiologist using pre-operative T_2_-w images. Following surgery, the surgeon aligned and marked the tumor for sectioning by an experienced soft-tissue sarcoma histopathologist. Distinct areas of different morphology on MRI were selected and ROIs (~1 cm^2^) from matched slices were selected jointly by the radiologist and histopathologist working in consensus (Figure 2). Anatomical landmarks on the specimen were used for matching ROIs. Up to 3 ROIs were chosen in each tumor to be representative of the tissues present, giving a total of 48 ROIs from 25 tumors.

Specimens were fixed in 10% formalin, placed in processing cassettes and processed using an automated tissue processor before embedding in paraffin. Sections 4 μm thick were cut using a Leitz sledge microtome, floated out on a water bath at 37°C, mounted onto positively charged, coated glass slides. Slides were then stored at room temperature. After dewaxing with xylene and rehydration through alcohols, they were stained using the standard techniques described as follows: slides were washed in water, stained with hematoxylin, rinsed in water, differentiated with 0.3% acid alcohol, rinsed again in water and stained with eosin for 2 min. They were dehydrated in xylene and mounted with mounting medium.

The following properties were assessed by the histopathologist in each ROI: cellularity, quantified using the nuclear-to-stromal ratio, which is defined as percentage of lesional nuclei to stromal tissue area present; fat fraction; and vessel density. ROIs were further categorized by stroma type as fibrous (with fibrous stroma grades 1–5), myxoid, and fibromyxoid. Correlation was assessed using Spearman's rank correlation coefficient (corr, Matlab 2016a). ROIs containing more than 80% fat were excluded from analysis of ADC, D, *f*, D^*^, and ${\text{R}}_{2}^{*}$.

## Results

### Imaging Parameters in the Whole Cohort and Individual Sarcoma Sub-Types

**Figure 3:**
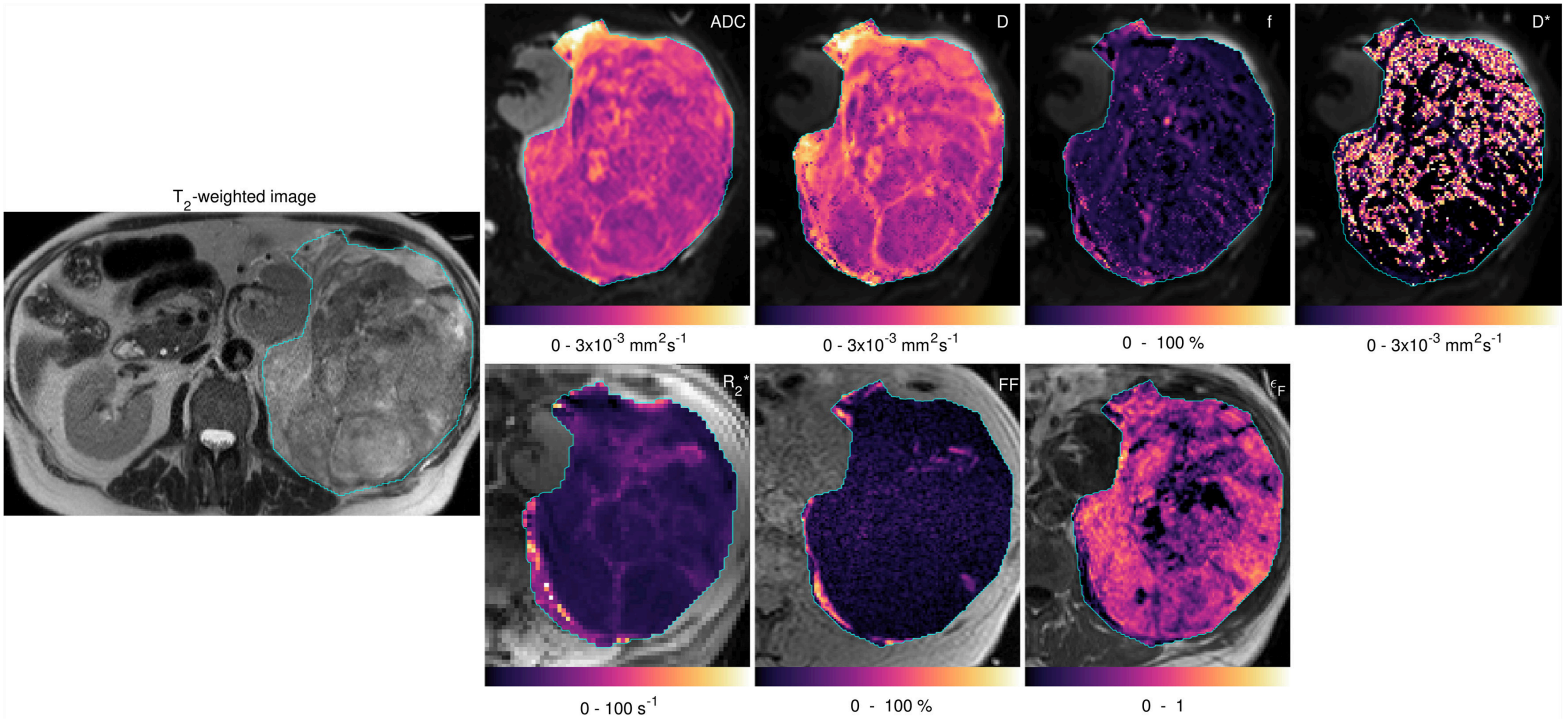
Acquired images and fitted/calculated parameters from the same slice as Figure 2. Left-hand image: T_2_-weighted image showing ROI. Right-hand panels: fitted/calculated values in ROI (color) overlaid on acquired images (gray scale). Top row: ADC overlaid on *b* = 50 s mm^−2^ image; D, *f*, and D* overlaid on *b* = 50 s mm^−2^ images. Bottom row: ${\text{R}}_{2}^{*}$ overlaid on TE = 5 ms gradient-echo image; FF overlaid on in-phase image; ε_F_ overlaid on pre-contrast image.

Figure 3 shows fitted parameters from the same slice as Figure 2. Wide ranges of each fitted parameter were observed across the cohort (Table 1), with median ADC estimates between 0.95 × 10^−3^ and 2.77 × 10^−3^ mm^2^ s^−1^ and a similar range of median D estimates (0.99 × 10^−3^ to 2.71 × 10^−3^ mm^2^ s^−1^); median ${\text{R}}_{2}^{*}$ estimates ranged from 5.19 to 58.27 s^−1^. Considering the two main sub-types separately, wide ranges of parameter estimates were observed within sub-types, for example median ADC between 0.95 × 10^−3^ and 2.77 × 10^−3^ mm^2^ s^−1^ for liposarcomas and between 1.06 × 10^−3^ and 1.76 × 10^−3^ mm^2^ s^−1^ for leiomyosarcomas. Wilcoxon rank sum tests did not show significant differences between sub-types for any of the quantitative MRI parameters studied (Table 1, *p* > 0.05), but there was a significant difference in tumor volumes (Table 1, *p* < 0.05). SNR in DW-MRI (*b* = 50 s mm^−2^) was 386.

**Table 1:** Baseline estimates of median ADC, IVIM parameters (D, *f*, D*), R_2_*, FF, EF, ε_F_, and volume for the cohort, and liposarcomas and leiomyosarcomas assessed separately.

**Parameter**	**All tumors (*n* = 30^**a**^)**	**Liposarcomas (*n* = 22^**a**^)**	**Leiomyosarcomas (*n* = 4^**a**^)**	***p*-value^**b**^**
ADC/10^−3^ mm^2^ s^−1^	1.70 (0.95–2.77)	1.85 (0.95–2.77)	1.31 (1.06–1.76)	0.08
D/10^−3^ mm^2^ s^−1^	1.65 (0.99–2.71)	1.77 (0.99–2.71)	1.26 (1.25–1.63)	0.2
*f/*%	6.85 (2.08–16.68)	6.37 (2.08–16.68)	12.78 (9.24–14.32)	0.06
D*/10^−3^ mm^2^ s^−1^	41.36 (14.69–85.28)	43.57 (14.69–85.28)	34.05 (15.14–42.67)	0.2
${\text{R}}_{2}^{*}$/s^−1^	18.50 (5.19–58.27)	19.29 (7.21–58.27)	16.84 (11.11–29.77)	0.6
Fat fraction (FF)/%	10.07 (5.25–85.09)	19.10 (6.30–85.09)	9.76 (9.17–10.36)	0.5
Enhancing fraction (EF)/%	91.03 (2.63–100.00)	83.92 (2.64–100.00)	97.68 (91.55–99.47)	0.06
Fractional enhancement (ε_F_)	0.34 (0.00–0.64)	0.29 (0.00–0.64)	0.40 (0.39–0.53)	0.09
Volume/cm^3^	1,002.30 (5.37–3,882.20)	1,584.80 (5.37–3,882.20)	53.54 (20.13–222.84)	0.01

*Table shows median values for cohort and sub-types. Values in brackets show ranges. Other summary statistics and repeatability are reported in Supplementary Table 2. In patients undergoing two baseline examinations, the mean of two estimates was used*.

a Numbers of patients: All tumors ADC n = 28, IVIM n = 26, R_2_* n = 27, FF n = 20, EF n = 29; liposarcomas ADC n = 20, IVIM n = 20, R_2_* n = 19, FF n = 14, EF n = 22; leiomyosarcomas ADC n = 4, IVIM n = 3, R_2_* n = 4, FF n = 2, EF n = 4.

b *p-values show results of Wilcoxon rank sum tests between liposarcomas and leiomyosarcomas*.

### Repeatability of Baseline Measurements

**Figure 4:**
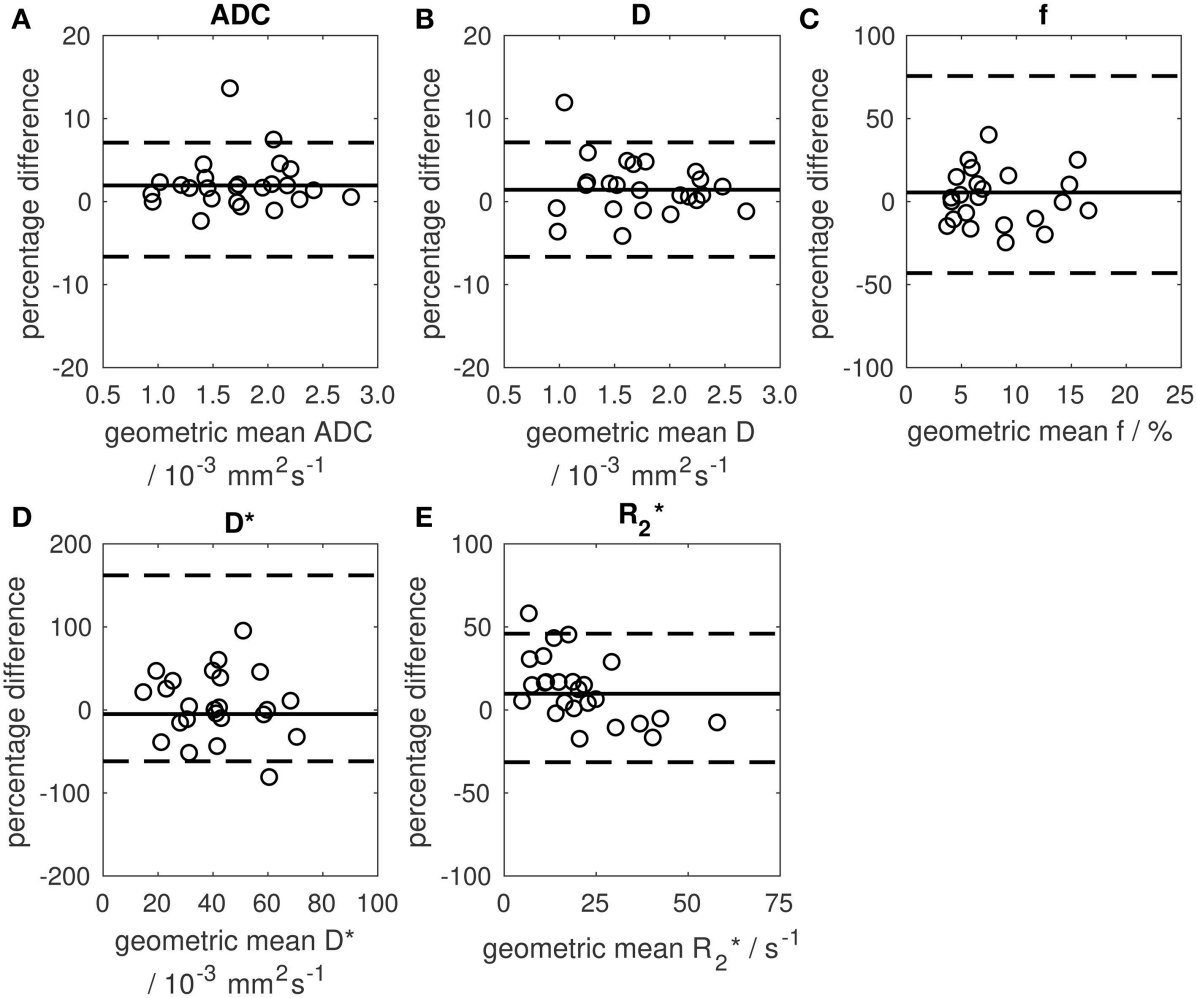
Bland-Altman plots showing percentage change between two baseline estimates of median parameter vs. their geometric mean. **(A)** median ADC, **(B)** median D, **(C)** median *f*, **(D)** median D*, **(E)** median ${\text{R}}_{2}^{*}$. Solid lines show the mean difference between two baseline examinations (mean differences were 1.9, 1.4, 5.5, −5.0, and 9.7% for ADC, D, *f*, D*, and ${\text{R}}_{2}^{*}$, respectively). Dashed lines show 95% limits of agreement.

Repeatability of median ADC was excellent with CoV = 2.5% and upper and lower 95% LoA 7.1 and −6.6%, respectively (Figure 4; Supplementary Table 2). Repeatability of mean ADC and other ADC centiles was also good (CoV = 2.5–4.4% for 10th, 25th, 75th, and 90th centiles) with poorer repeatability of standard deviation (CoV = 12.3%). Repeatability of D was similar to ADC, but repeatability of *f* and D^*^ was poor (CoVs of 2.5, 20.5, and 35.8% for median D, *f*, and D^*^, respectively). Repeatability of ${\text{R}}_{2}^{*}$ was poorer than for ADC (CoV = 13.7% for median ${\text{R}}_{2}^{*}$).

### Post-radiotherapy Changes

**Figure 5:**
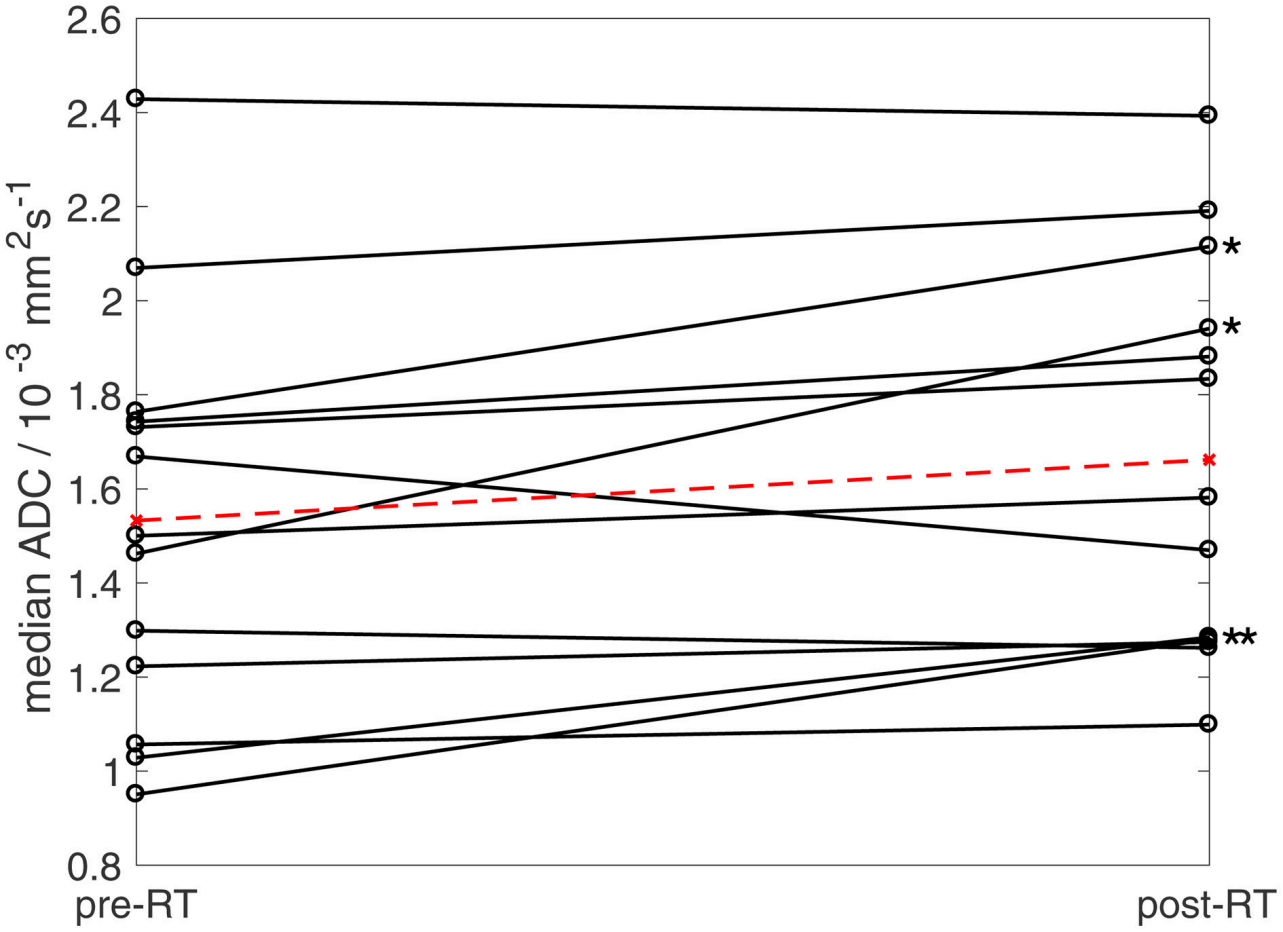
Median ADC estimates pre- and post-radiotherapy. Ladder plot showing median ADC estimates for 13 patients pre- and post-radiotherapy (solid lines). In patients undergoing two baseline examinations, the mean of two estimates was used. Dashed line shows mean values for the cohort. Asterisks show four patients exhibiting post-treatment increases in median ADC outside baseline 95% LoA (One patient who underwent radiotherapy was excluded from ADC analysis as the tumor [well-differentiated liposarcoma] was composed of more than 80% fat and was therefore not evaluable using fat-suppressed diffusion-weighted imaging).

All patients were assessed by RECIST 1.1 criteria as having stable disease post-radiotherapy (*n* = 14). However, a significant increase in median ADC was observed in the cohort (Figure 5, Wilcoxon signed rank test, *p* = 0.02). Considering patients individually, four tumors (one synovial sarcoma, one dedifferentiated liposarcoma, one leiomyosarcoma, one pleomorphic sarcoma not otherwise specified) exhibited a post-radiotherapy increase in median ADC outside the 95% LoA, indicating a post-treatment change outside the expected variation of repeated measurements with 95% confidence (Figure 5, Supplementary Table 3). Cohort assessments also showed significant increases in the mean, standard deviation, 25th, 75th, and 90th centiles of ADC (Wilcoxon signed rank test, *p* < 0.05), and in the mean, 75th and 90th centiles of D (*p* < 0.05), but no significant post-radiotherapy changes were observed in *f*, D^*^, ${\text{R}}_{2}^{*}$, FF, EF, or ε_F_ (*p* > 0.05). The majority of tumors (10/14) increased in volume following radiotherapy (median volume change +4%, range −10 to +31%). No correlation was observed between volume changes and post-treatment changes in any of the quantitative MRI parameters studied.

### Histopathological Correlation

**Figure 6:**
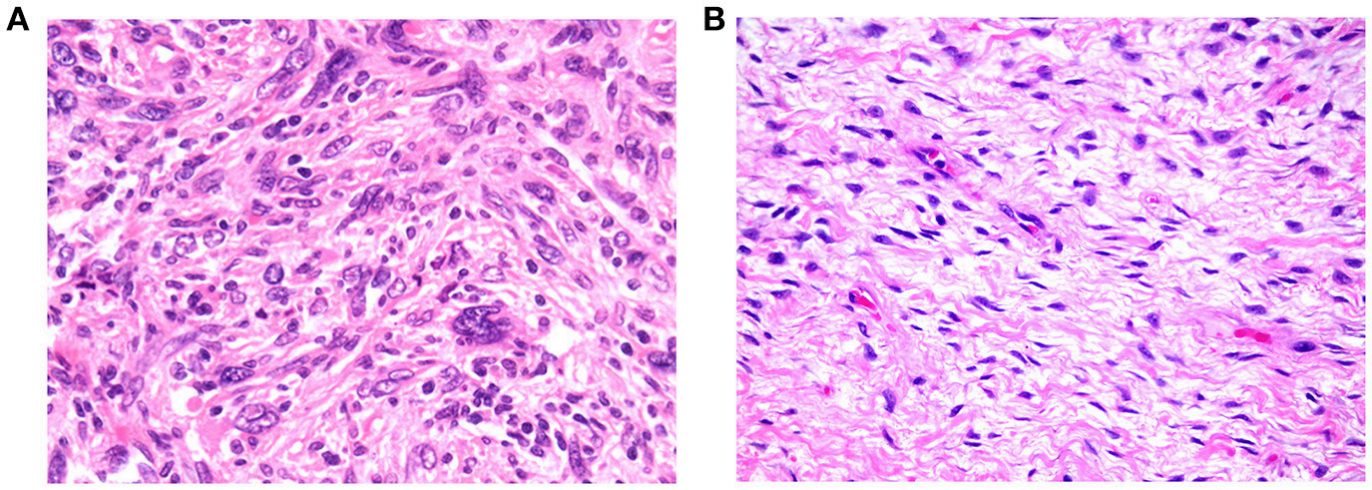
**(A)** Example of tumor exhibiting high cellularity, with patternless distributions of markedly pleomorphic cells dispersed in moderate amounts of collagenous stroma (200× magnification). **(B)** Example of tumor exhibiting low cellularity, comprising loose fascicles of relatively bland spindle cells, dispersed in abundant collagenous stroma (200× magnification).

**Figure 7:**
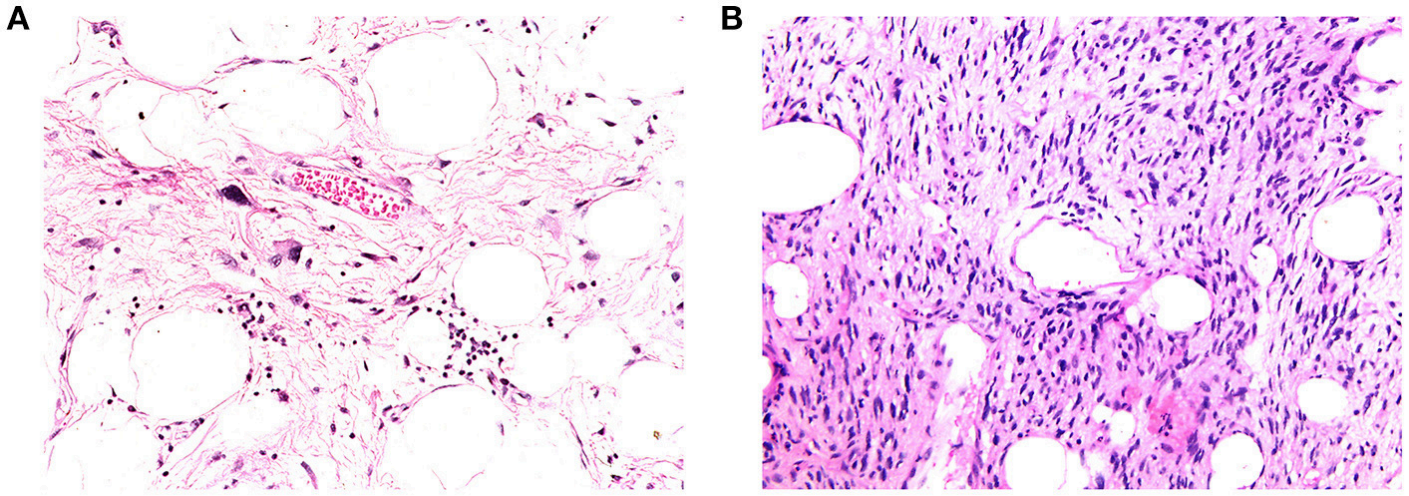
**(A)** Example of high-fat fraction tumor showing prominent lobules and sheets of adipocytes, intersected by sparsely cellular fibrous septa. Occasional atypical hyperchromatic nuclei are apparent within the fibrous stroma (400× magnification). **(B)** Example of low-fat fraction tumor largely composed of prominent spindle cells arising in loose fascicles within delicately collagenous stroma. Only small numbers of adipocytes are scattered within the neoplasm (100× magnification).

**Figure 8:**
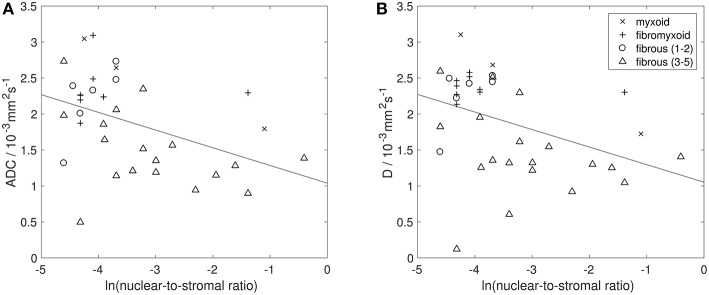
Histopathological assessment of cellularity. Natural logarithm of nuclear-to-stromal ratio (estimated from histopathological analysis) vs. **(A)** apparent diffusion coefficient (ADC, estimated from DW-MRI) and **(B)** diffusion coefficient (D, from IVIM model of DW-MRI). Each point represents one ROI. Solid black line shows line of best fit. Points are labeled by histopathological assessment of stroma type (myxoid, fibromyxoid, or fibrous), with fibrous stroma types labeled by stroma grade (lower grades 1–2, and higher grades 3–5). ROIs that consisted of more than 80% fat were excluded from analysis of ADC and D.

**Figure 9:**
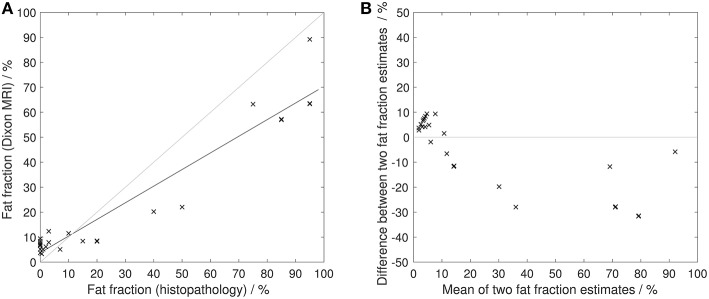
Histopathological assessment of fat fraction. **(A)** Fat fraction estimated from Dixon MRI vs. fat fraction estimated from histopathological assessment. Solid black line shows line of best fit. Gray line shows line of identity. **(B)** Difference between fat fraction estimated from Dixon MRI and fat fraction estimated from histopathological assessment, plotted against the mean of the two measurements. Gray line shows line of no difference between two measurements.

Figures 6, 7 show examples of tumors with high/low cellularity and high/low fat fraction, respectively. Figure 8A demonstrates negative correlation between ADC and nuclear-to-stromal ratio, with high ADCs in ROIs with low nuclear-to-stromal ratio and low ADCs in ROIs with high nuclear-to-stromal ratio (*r* = −0.42, *p* = 0.01, 95% confidence interval (CI) −0.22 to −0.59). Figure 8A also shows a dependence of ADC on stroma type and stroma grade. In fibrous stroma, higher ADCs were observed in fibrous grades 1 and 2, with lower ADCs in fibrous grades 3–5 (Wilcoxon rank sum test, *p* = 0.01). Myxoid and fibromyxoid stroma also exhibited high ADCs. Figure 8B shows similar dependence of D on nuclear-to-stromal ratio (*r* = −0.45, *p* = 0.005, 95% CI −0.25 to −0.62), and stroma type, and stroma grade. There was no significant difference in ADC, D, or nuclear-to-stromal ratio between post-radiotherapy and surgery-only cohorts. FF estimated from Dixon MRI showed strong correlation with histopathological assessment (Figure 9A, *r* = 0.79, *p* = 10^−7^, 95% CI 0.69–0.86). Estimates from Dixon MRI were slightly higher than histopathological assessment at low FF, but lower than histopathological assessments at high FF (Figures 9A,B). There was no clear relationship between histopathological assessment of vessel density and either *f*, *f* D^*^, ${\text{R}}_{2}^{*}$, EF, or ε_F_, in the whole cohort nor in post-radiotherapy or surgery-only cohorts assessed separately.

## Discussion

The baseline estimates of quantitative MRI parameters reported in this cohort of 30 patients, together with their repeatability, provide essential information for planning multi-parametric imaging studies in soft-tissue sarcomas, including clinical trials of new therapies. The significant post-treatment changes in ADC suggest that ADC is a useful biomarker for response assessment in soft-tissue sarcomas. However, the wide ranges of baseline ADCs and high ADCs in many tumors at baseline should be considered when characterizing tumors and assessing response. Previous studies have suggested that a two-point scheme should employ an upper b-value of 1.1/ADC (9), suggesting that b-values of 397 and 1,158 s mm^−2^ would be appropriate for ADCs of 0.95 × 10^−3^ and 2.77 × 10^−3^ mm^2^ s^−1^, respectively. In a clinical study, however, a compromise may be required to accommodate the range of ADCs expected within the study. The mixture of sarcoma sub-types included in this study, which is typical of trials in this rare tumor type, showed that wide ranges quantitative MRI parameters are also present within sub-types and must still be taken into account in studies with more restricted inclusion criteria.

**Figure 10:**
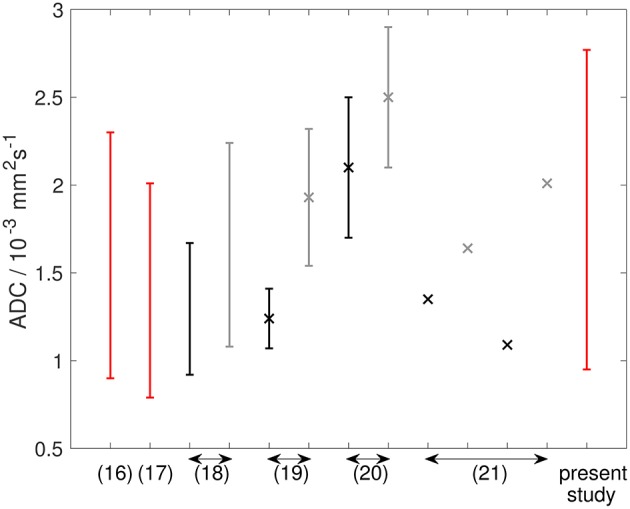
Graph showing ADC data from the literature (described in Table 2), alongside data from the present study. References are shown on the x-axis. Double-headed arrows show multiple data points from the same reference. Red lines show ADC estimates from soft-tissue sarcomas at baseline. Black and gray lines show ADC estimates from osteosarcomas [and Ewing sarcomas in Hayashida et al. (21)] at baseline and post-treatment, respectively. Studies reporting mean ± standard deviation are shown as markers (×) with error bars representing standard deviation. Studies reporting only an average value are shown as markers (×) without error bars. Studies reporting a range are shown as error bars (upper and lower ends of range) without markers.

The ADCs in this study are in broad agreement with other soft-tissue sarcoma studies although the wider range and higher ADCs reported here may reflect the mixtures of sarcoma sub-types (Table 2 and Figure 10). ADCs in soft-tissue sarcomas are notably higher than other tumor types, including osteosarcomas, which highlights the importance of establishing ranges of quantitative MRI parameters in soft-tissue sarcomas (Table 2 and Figure 10). Response thresholds established in other tumors may also differ (22).

**Table 2:** ADC estimates reported in previous studies of soft-tissue sarcoma and osteosarcoma.

**Tumors**	**Patients**	**ADC estimates**
Soft-tissue sarcomas (mixed sub-types) in trunk and limbs	13	Mean ADCs between 0.9 × 10^−3^ mm^2^ s^−1^ and 2.3 × 10^−3^ mm^2^ s^−1^ in pre-treatment measurements (16)
Soft-tissue sarcomas (mixed sub-types) in trunk, limbs, and head	23	Mean ADCs between 0.79 × 10^−3^ mm^2^ s^−1^ and 2.01 × 10^−3^ mm^2^ s^−1^ in pre-treatment measurements (17)
Osteosarcoma	31	Mean ADCs between 0.92 × 10^−3^ mm^2^ s^−1^ and 1.67 × 10^−3^ mm^2^ s^−1^ at baseline and between 1.08 × 10^−3^ mm^2^ s^−1^ and 2.24 × 10^−3^ mm^2^ s^−1^ after chemotherapy (18)
Osteosarcoma	35	Mean ADCs (1.24 ± 0.17) × 10^−3^ mm^2^ s^−1^ at baseline and (1.93 ± 0.39) × 10^−3^ mm^2^ s^−1^ after chemotherapy (19)
Osteosarcoma (pediatric)	8	Mean ADCs (2.1 ± 0.4) × 10^−3^ mm^2^ s^−1^ at baseline and (2.5 ± 0.4) × 10^−3^ mm^2^ s^−1^ after chemotherapy (20)
Osteosarcoma and Ewing sarcoma	18	Mean ADCs 1.35 × 10^−3^ mm^2^ s^−1^ at baseline and 1.64 × 10^−3^ mm^2^ s^−1^ after chemotherapy in tumors with < 90% necrosis post-treatment; mean ADCs 1.09 × 10^−3^ mm^2^ s^−1^ at baseline and 2.01 × 10^−3^ mm^2^ s^−1^ after chemotherapy in tumors with more than 90% necrosis post treatment (21)

The excellent repeatability, particularly median and mean ADC, indicates that ADC is a robust metric in clinical studies in retroperitoneal sarcomas. ADC repeatability was better than in some other extra-cranial soft-tissue tumors, where CoVs up to 7% have been observed (23). Retroperitoneal sarcomas also exhibit good repeatability of other ADC centile values (10th to 90th centiles), in agreement with studies in other solid tumors (24). Good baseline repeatability confers high sensitivity to post-treatment changes, as demonstrated by the significant ADC increase post-radiotherapy. A post-treatment increase of 7.1% in median ADC would be outside the upper 95% LoA, indicating a change outside the expected variation of repeated measurements.

The correlation between restricted diffusion (low ADC or D) and high cellularity (high nuclear-to-stromal ratio) demonstrates that the degree of restricted diffusion relates to the density of tumor cells. A similar relationship between ADC and cellularity was observed previously in soft-tissue sarcomas (25). However, the present study suggests that the relationship is more complicated than a simple correlation owing to the differences in ADC or D between stroma types and stroma grades. ADCs of myxoid and fibromyxoid regions are high compared with other tumor types, while low ADCs may be indicative of fibrous regions.

The increase in ADC post-radiotherapy agrees with other studies (16) and was significant, although behavior across the cohort was mixed, which may reflect the mixture of tumor sub-types. Double-baseline measurements enable identification of significant post-treatment changes in individuals, showing that ADC was able to reflect radiotherapy response despite stable disease categorization by RECIST 1.1.

Although D exhibited similar repeatability to ADC, the repeatability of other IVIM parameters (*f*, D^*^) was poorer, in agreement with previous studies (24, 26–28). IVIM parameters did not contribute additional information on post-treatment changes, since D provided similar information to ADC (29), while *f* and D^*^ did not change significantly post-treatment. Estimates of *f* were lower than in other tissues where the IVIM model has been more widely applied (26). The attenuation of the DW-MRI signal with increasing diffusion weighting did not exhibit the steep deviation from mono-exponential behavior at low diffusion-weightings that is characterized by the IVIM model, and no correlation was observed between vessel density and *f* or *f* D^*^, suggesting that this model may not describe perfusion and diffusion components of the DW-MRI signal in these tumors. The difficulty of fitting a bi-exponential model at low perfusion fractions has also been explored in other studies (30).

The poorer repeatability of ${\text{R}}_{2}^{*}$ compared with ADC and D agrees with previous studies of pelvic (31), prostate (32), and head-and-neck tumors (33). The poor repeatability and absence of post-radiotherapy changes suggest ${\text{R}}_{2}^{*}$ may be of limited value for response assessment in a clinical setting in soft-tissue sarcomas.

FF was not useful for detecting post-treatment changes but the large range of baseline FF highlights the presence of fatty components in soft-tissue sarcomas. The absence of any significant difference in FF between liposarcomas and leiomyosarcomas may be due to dedifferentiated components in most of the liposarcomas. Strong correlation between FF from Dixon MRI and histopathology demonstrates the value of MRI in quantifying fat, which may be valuable in distinguishing fat in low-grade liposarcomas and quantifying well and dedifferentiated elements. FF was estimated from signal intensities in fat and water images reconstructed using the manufacturer's Dixon algorithm. Proton density-weighted imaging was used to minimize T_1_-related bias (34) but noise bias may contribute to errors, particularly at very high and low FF (35).

Baseline EF estimates ranged from strongly enhancing to largely non-enhancing tumors, reflecting the inter-tumor heterogeneity. No significant post-radiotherapy change was observed in EF. DCE-MRI has been shown to be indicative of response in smaller tumors (6) but whole-tumor assessments of EF were employed here as large volume coverage limited the temporal resolution for pharmacokinetic modeling. The absence of correlation between histopathological assessment of vessel density and MRI parameters relating to vascular properties (*f*, *f* D^*^, ${\text{R}}_{2}^{*}$, EF) may arise since these parameters also relate to functional properties of the vasculature, such as flow, oxygenation and permeability.

The functional imaging parameters described here characterize components of these highly variable tumors. The repeatability and relation to histopathology suggest that functional imaging parameters can be incorporated confidently as secondary end-points in clinical trials. Development of methods to quantify heterogeneous post-treatment changes may be valuable in soft-tissue sarcomas.

There were limitations to the study. Firstly, only 30 patients were recruited but, nevertheless, significant post-radiotherapy changes were detected. Secondly, the small numbers of rare sub-types, which is typical of many sarcoma trials, precluded separate sub-type assessments; the comparison between liposarcomas and leiomyosarcomas is also limited by small sample sizes. Thirdly, it was not possible to assess repeatability of EF at a second baseline on the same day. It was therefore possible to assess cohort changes in EF, but not individual post-treatment changes. Fourthly, the strong correlation between FF from Dixon MRI and histopathology may also arise from the high numbers of samples with very high and very low FF; larger numbers of samples across the range of FF are, therefore, required to fully assess agreement between FF estimates. Finally, there was a degree of subjectivity in matching ROIs in MR images to histology samples, which introduces some uncertainty in the MRI-histopathology correlation.

In conclusion, the wide ranges of ADCs in retroperitoneal soft-tissue sarcomas reflect intra- and inter-tumor heterogeneity. ADCs are higher than in other soft-tissue tumors. ADCs exhibit excellent baseline repeatability, and can detect response by identifying post-treatment changes >7.1%. ADC increased significantly post-radiotherapy in a mixed cohort of retroperitoneal soft-tissue sarcomas and significant individual responses were detectable in disease classed as stable by RECIST 1.1. ADC and D correlate with cellularity, stroma type, and stroma grade, and Dixon estimates of fat fraction show strong correlation with tissue composition.

## Data Availability

The data from the present study are available on a cancer imaging research repository (https://xnatcruk.icr.ac.uk/XNAT_CRUK_ARCHIVE). Access requests will be granted depending on appropriate regulatory and institutional approvals upon contacting the corresponding author.

The datasets generated for this study are available on request to the corresponding author.

## Ethics Statement

This study was carried out in accordance with the recommendations of the Royal Marsden Hospital Committee for Clinical Research and approval from a national Research Ethics Committee (East of England—Cambridge East Research Ethics Committee). All subjects gave written informed consent in accordance with the Declaration of Helsinki (Clinical trials registry: ClinicalTrials.gov, registration number: NCT01902667).

## Author Contributions

CM, AM, DS, DC, KT, ML, AH, DH, and NdS devised the study. CM, AM, DS, DC, KT, AH, DH, JW, MS, SG, VM, EM, SZ, and DH contributed to data acquisition/collection. JW and CM performed the analysis. All authors contributed to manuscript revision and approved the final version.

### Conflict of Interest Statement

The authors declare that the research was conducted in the absence of any commercial or financial relationships that could be construed as a potential conflict of interest.

## Abbreviations

ADC: apparent diffusion coefficient
CI: confidence interval
CoV: coefficient of variation
CT: computed tomography
CTV: clinical target volume
D: diffusion coefficient
D^*^: pseudo-diffusion coefficient
DCE-MRI: dynamic contrast-enhanced magnetic resonance imaging
DW-EPI: diffusion-weighted echo-planar imaging
DW-MRI: diffusion-weighted magnetic resonance imaging
EF: enhancing fraction
*f*: volume fraction
FF: fat fraction
GTV: gross tumor volume
IMRT: intensity-modulated radiotherapy
IVIM: intra-voxel incoherent motion
LoA: limit of agreement
MRI: magnetic resonance imaging
PACS: picture archiving and communication system
PTV: planning target volume
SNR: signal to noise ratio
${\text{R}}_{2}^{*}$: transverse relaxation rate
RECIST 1. 1: response evaluation criteria in solid tumors (version 1.1)
ROI: region of interest
VOI: volume of interest.
