# Supplementary material for: Utility of Multi-Parametric Quantitative Magnetic Resonance Imaging for Characterization and Radiotherapy Response Assessment in Soft-Tissue Sarcomas and Correlation With Histopathology
Source: Front Oncol. 2019 Apr 25;9:280. doi: 10.3389/fonc.2019.00280 (PMC6494941; doi:10.3389/fonc.2019.00280)
Supplement: Supplementary file 1 [file Table_1.DOCX]

Supplementary Material

# Supplementary Table 1

**Supplementary Table 1.** Imaging sequence parameters. *FLASH* Fast low-angle shot, *GRAPPA* generalized autocalibrating partially parallel acquisitions, *CAIPIRINHA* controlled aliasing in parallel imaging results in higher acceleration, *SPAIR* spectral adiabatic inversion recovery.

^a^ Diffusion-weighted EPI sequences used bipolar diffusion-encoding gradients. The durations and separations of the four diffusion gradient lobes are described using notation adapted from Koch M and Norris DG. *Phys. Med. Biol.* (2000) 45:3821–3832, where

d_n_ = duration of n^th^ diffusion gradient lobe (measured from start of gradient to end of flat top, before rampdown);

a_n_ = time between n^th^ and (n+1)^th^ diffusion gradient lobes (measured from end of flat top to start of next gradient);

Δ = ‘effective diffusion time’, defined as interval between start of first diffusion gradient lobe and start of third diffusion gradient lobe.

|  | **DW-MRI** | **IVIM** | **Multiple gradient echo imaging** | **Dixon** | **Pre- and post-Gd T_1_-weighted imaging** |
| --- | --- | --- | --- | --- | --- |
| Quantitative MRI parameter estimated | ADC | D, *f*, D* | R_2_* | FF | EF, ε_F_ |
| Sequence | ss-EPI | ss-EPI | 2D FLASH | 3D FLASH | 3D FLASH |
| Slice orientation | Axial | Axial | Axial | Axial | Axial |
| PE direction | AP | AP | AP | AP (in-plane) | AP (in-plane) |
| FOV / mm x mm | 420 x 336 | 380 x 308 | 420 x 341 | 380 x 285 | 380 x 285 |
| Acquired matrix | 160 x 128 | 128 x 104 | 128 x 104 | 256 x 144 | 256 x 144 |
| Slice thickness / mm | 5 | 6 | 6 | 5 | 5 |
| Slice gap / mm | 0 | 1.2 | 1.2 | 0 | 0 |
| Number of slices | 40 slices per station (>1 station if necessary to cover tumour) | 25 slices (centred on central slice of tumour) | 7 slices (centred on central slice of tumour) | 40 slices per slab (>1 station if necessary to cover tumour) | 40 slices per slab (>1 station if necessary to cover tumour) |
| Echo time (TE) / ms | 65 | 75 | 5, 10, 30, 45, 55 | 2.39 and 4.77 | 1.1 |
| Repetition time (TR) / ms | 9200 | 5200 | 193 | 7.05 | 3.8 |
| Nutation angle / ° | 90 | 90 | 35 | 3 | 17 |
| Receiver bandwidth /  Hz/pixel | 1955 | 1700 | 260 | 400 | 250 |
| Number of signal averages (NSA) | NSA = 4 for b = 50 s mm^-2^ and b = 600 s mm^-2^; NSA = 5 for b = 900 s mm^-2^ | 3 | 1 | 1 | 1 |
| Reduced acquisition | GRAPPA, reduction factor 2 | GRAPPA, reduction factor 2 | GRAPPA, reduction factor 2 | CAIPIRINHA, acceleration factor 2x2 | none |
| Fat suppression | SPAIR | SPAIR | Water-only excitation | none | none |
| Breathing instructions | Free breathing | Free breathing | Four breath-holds on expiration | Breath-hold on expiration | Breath-hold on expiration |
| Diffusion weightings /  s mm^-2^ | 50, 600, 900 | 0, 25, 50, 80, 150, 300, 500, 800 | n/a | n/a | n/a |
| Diffusion encoding scheme | Three-scan trace  (gradient directions [1.0, 1.0, -0.5], [1.0, -0.5, 1.0], [-0.5, 1.0, 1.0]) | Three-scan trace  (gradient directions [1.0, 1.0, -0.5], [1.0, -0.5, 1.0], [-0.5, 1.0, 1.0]) | n/a | n/a | n/a |
| Diffusion gradient timings / ms ^a^ | d_1_ = 5.3; d_2_ = 12.1; d_3_ = 13.7; d_4_ = 3.7;  a_1_ = 8.0; a_2_ = 0.6; a_3_ = 6.0; Δ = 26.0 | d_1_ = 5.4; d_2_ = 12.6; d_3_ = 14.6; d_4_ = 3.5;  a_1_ = 9.8; a_2_ = 0.6; a_3_ = 9.8;  Δ = 28.4 | n/a | n/a | n/a |
| Acquisition time / mins:secs | 6:28 | 5:59 | 0:54 | 0:14 | 0:22 |
| Contrast agent | n/a | n/a | n/a | n/a | Post-contrast images acquired 4 minutes post-injection (Dotarem, 0.2ml/kg body-weight, 2ml/sec). |
