# Supplementary material for: Utility of Multi-Parametric Quantitative Magnetic Resonance Imaging for Characterization and Radiotherapy Response Assessment in Soft-Tissue Sarcomas and Correlation With Histopathology
Source: Front Oncol. 2019 Apr 25;9:280. doi: 10.3389/fonc.2019.00280 (PMC6494941; doi:10.3389/fonc.2019.00280)
Supplement: Supplementary file 2 [file Table_2.DOCX]

Supplementary Material

# Supplementary Table 2

**Supplementary Table 2.** Baseline estimates of ADC, D, *f*, D*, and R_2_*. For each fitted parameter, summary statistics (median, mean, standard deviation, 10^th^, 25^th^, 75^th^, 90^th^ centiles, skew, and kurtosis) were evaluated from all fitted pixels in each VOI. The median and range across all patients are reported for each summary statistic. In patients undergoing two baseline examinations, the mean of two estimates was used. (Median estimates of each fitted parameter were also reported in Table 2 in the main document but are included here for completeness.)

^a^ Repeatability is described using 95% limits of agreement (LoA) estimated using repeated baseline measurements. Repeatability of the median, mean, standard deviation, 10^th^, 25^th^, 75^th^, and 90^th^ centiles was assessed using the natural logarithm of the values and is reported on a percentage scale; coefficients of variation (CoV) are also reported for these summary statistics to facilitate comparison with other studies. 95% LoA of skew and kurtosis were estimated using the untransformed values and are reported as absolute changes. 95% confidence intervals for LoA and CoV are shown in brackets.

^b^ Repeated baseline measurements for ADC repeatability assessments were acquired in 25 patients, and in 24 patients for D, *f*, D* estimates, and 25 patients for R_2_* estimates.

| Parameter | Summary statistic | Median (range) | 95 % LoA | | CoV / %  ^a^ |
| --- | --- | --- | --- | --- | --- |
|  |  |  | Upper LoA / % ^a^ | Lower LoA / % ^a^ |  |
| ADC  /10^-3^mm^2^s^-1^ (n = 28 ^b^) | median | 1.70  (0.95 to 2.77) | 7.1  (5.5, 9.9) | -6.6  (-9.0, -5.2) | 2.5  (1.9, 3.4) |
|  | mean | 1.74  (1.01 to 2.41) | 7.8  (6.0, 10.9) | -7.2  (-9.8, -5.7) | 2.7  (2.1, 3.7) |
|  | standard deviation | 0.32  (0.16 to 0.95) | 40.5  (30.5, 59.8) | -28.8  (-37.4, -23.4) | 12.3  (9.6, 17.0) |
|  | 10^th^ centile | 1.18  (0.78 to 2.17) | 12.9  (10.0, 18.2) | -11.4  (15.4, -9.1) | 4.4  (3.4, 6.0) |
|  | 25^th^ centile | 1.36  (0.85 to 2.30) | 8.4  (6.6, 11.8) | -7.8  (-10.6, -6.2) | 2.9  (2.3, 4.0) |
|  | 75^th^ centile | 1.85  (1.11 to 3.06) | 7.1  (5.5, 9.9) | -6.6  (-9.0, -5.2) | 2.5  (1.9, 3.4) |
|  | 90^th^ centile | 2.04  (1.23 to 3.20) | 7.2  (5.6, 10.0) | -6.7  (-9.1, -5.3) | 2.5  (2.0, 3.4) |
|  | skew | 0.23  (-1.58 to 2.92) | 0.61  (0.48, 0.85) | -0.61  (-0.85, -0.48) | - |
|  | kurtosis | 4.22  (1.39 to 14.90) | 3.26  (2.56, 4.50) | -3.26  (-4.50, -2.56) | - |
| D  /10^-3^mm^2^s^-1^ (n = 26 ^b^) | median | 1.65 (0.99 to 2.71) | 7.1  (5.5, 10.1) | -6.7  (-9.1, -5.2) | 2.5  (1.9, 3.5) |
|  | mean | 1.63 (1.00 to 2.47) | 6.4  (4.9, 9.0) | -6.0  (-8.2, -4.7) | 2.2  (1.7. 3.1) |
|  | standard deviation | 0.36 (0.20 to 1.03) | 26.8  (20.3, 39.1) | -21.1  (-28.1, -16.9) | 8.6  (6.7, 11.9) |
|  | 10^th^ centile | 1.19 (0.44 to 2.23) | 19.3  (14.7, 27.8) | -16.2  (-21.7, -12.9) | 6.4  (5.0, 8.9) |
|  | 25^th^ centile | 1.35 (0.70 to 2.35) | 8.1  (6.2, 11.4) | -7.5  (-10.2, -5.9) | 2.8  (2.2, 3.9) |
|  | 75^th^ centile | 1.94 (1.17 to 3.08) | 6.5  (5.0, 9.1) | -6.1  (-8.4, -4.8) | 2.3  (1.8, 3.1) |
|  | 90^th^ centile | 2.12 (1.39 to 3.45) | 6.2  (4.8, 8.7) | -5.8  (-8.0, -4.6) | 2.2  (1.7, 3.0) |
|  | skew | 0.11 (-2.56 to 1.83) | 0.78  (0.61, 1.09) | -0.78  (-1.09, -0.61) | - |
|  | kurtosis | 5.19 (1.49 to 15.17) | 2.86  (2.23, 3.97) | -2.86  (-3.97, -2.23) | - |
| *f* / %  (n = 26 ^b^) | median | 6.85 (2.08 to 16.68) | 75.6  (55.2, 118.9) | -43.1  (-54.3, -35.6) | 20.5  (16.0, 28.8) |
|  | mean | 9.90 (4.32 to 20.36) | 36.4  (27.4, 54.0) | -26.7  (-35.1, -21.5) | 11.2  (8.8, 15.7) |
|  | standard deviation | 9.14 (4.97 to 17.50) | 29.2  (22.2, 42.8) | -22.6  (-30.0, -18.1) | 9.3  (7.2, 12.9) |
|  | 10^th^ centile | 0.31 (0.03 to 7.67) | 1240.8  (659.0, 3601.0) | -92.5  (-97.3, -86.8) | 118.5  (84.1, 211.2) |
|  | 25^th^ centile | 3.42 (0.21 to 11.09) | 523.1  (317.3, 1174.5) | -84.0  (-92.2, -76.0) | 73.9  (55.2, 115.0) |
|  | 75^th^ centile | 13.07 (5.68 to 29.84) | 32.4  (24.5, 47.7) | -24.5  (-32.3, -19.7) | 10.1  (7.9, 14.1) |
|  | 90^th^ centile | 22.83 (9.55 to 46.55) | 32.3  (24.5, 47.7) | -24.4  (-32.3, -19.7) | 10.1  (7.9, 14.1) |
|  | skew | 2.15 (0.74 to 4.17) | 0.96  (0.75, 1.34) | -0.96  (-1.34, -0.75) | - |
|  | kurtosis | 9.48 (3.20 to 35.30) | 8.85  (6.91, 12.31) | -8.85  (-12.31, -6.91) | - |
| D*  /10^-3^mm^2^s^-1^ (n = 26 ^b^) | median | 41.36 (14.69 to 85.28) | 162.1  (112.2, 282.1) | -61.9  (-73.8, -52.9) | 35.8  (27.7, 51.3) |
|  | mean | 204.75 (59.92 to 268.97) | 54.7  (40.6, 83.5) | -35.4  (-45.5, -28.9) | 15.8  (12.3, 22.2) |
|  | standard deviation | 286.52 (125.84 to 313.82) | 20.3  (15.6, 29.4) | -16.9  (-22.7, -13.5) | 6.7  (5.2, 9.3) |
|  | 10^th^ centile | 7.78 (6.22 to 13.00) | 43.1  (32.3, 64.7) | -30.1  (-39.3, -24.4) | 13.0  (10.1, 18.1) |
|  | 25^th^ centile | 14.31 (8.45 to 25.76) | 84.2  (61.1, 133.9) | -45.7  (-57.2, -37.9) | 22.3  (17.3, 31.4) |
|  | 75^th^ centile | 323.13 (51.16 to 510.66) | 234.3  (156.6, 435.9) | -70.1  (-81.3, -61.0) | 45.7  (35.0, 66.6) |
|  | 90^th^ centile | 720.92 (80.46 to 801.90) | 31.5  (23.9, 46.4) | -24.0  (-31.7, -19.3) | 9.9  (7.7, 13.8) |
|  | skew | 1.40 (0.92 to 5.18) | 0.81  (0.63, 1.12) | -0.81  (-1.12, -0.63) | - |
|  | kurtosis | 3.57 (2.40 to 31.17) | 2.83  (2.21, 3.94) | -2.83  (-3.94, -2.21) | - |
| R_2_* / s^-1^  (n = 27 ^b^) | median | 18.50 (5.19 to 58.27) | 45.9  (34.5, 68.5) | -31.5  (-40.6, -25.6) | 13.7  (10.7, 19.0) |
|  | mean | 21.25 (6.25 to 79.89) | 44.6  (33.6, 66.5) | -30.9  (-39.9, -25.1) | 13.4  (10.5, 18.5) |
|  | standard deviation | 17.20 (4.27 to 124.98) | 131.0  (92.8, 217.6) | 56.7  (-68.5, -48.1) | 30.9  (24.0, 43.6) |
|  | 10^th^ centile | 9.57 (1.26 to 34.06) | 48.7  (36.5, 72.8) | -32.7  (-42.1, -26.7) | 14.4  (11.3, 19.9) |
|  | 25^th^ centile | 12.15 (2.74 to 44.75) | 45.1  (33.9, 67.2) | -31.1  (-40.2, -25.3) | 13.5  (10.6, 18.7) |
|  | 75^th^ centile | 24.38 (6.66 to 96.73) | 56.1  (41.8, 84.9) | -35.9  (-45.9, -29.5) | 16.2  (12.7, 22.5) |
|  | 90^th^ centile | 32.62 (9.21 to 205.75) | 50.1  (37.5, 75.1) | -33.4  (-42.9, -27.3) | 14.7  (11.5, 20.4) |
|  | skew | 5.50 (2.47 to 30.61) | 19.55  (15.33, 26.98) | -19.55  (-26.98, -15.33) | - |
|  | kurtosis | 60.56 (10.34 to 1.87×10^3^) | 1265.56  (992.53, 1746.99) | -1265.56  (-1746.99, -992.53) | - |
