# Supplementary material for: Utility of Multi-Parametric Quantitative Magnetic Resonance Imaging for Characterization and Radiotherapy Response Assessment in Soft-Tissue Sarcomas and Correlation With Histopathology
Source: Front Oncol. 2019 Apr 25;9:280. doi: 10.3389/fonc.2019.00280 (PMC6494941; doi:10.3389/fonc.2019.00280)
Supplement: Supplementary file 3 [file Table_3.DOCX]

Supplementary Material

# Supplementary Table 3

**Supplementary Table 3.** Numbers of patients where an increase or decrease was observed that was outside the 95% LoA**.** For each parameter, numbers of patients are reported as a proportion of patients evaluated. Changes in summary statistics that were within the 95% CI on the LoA are not included.

| Parameter | Summary statistic | Number of patients showing increase after radiotherapy | Number of patients showing decrease after radiotherapy |
| --- | --- | --- | --- |
| ADC  /10^-3^mm^2^s^-1^ | median | 4/13 | 1/13 |
|  | mean | 4/13 | 1/13 |
|  | standard deviation | 0/13 | 0/13 |
|  | 10^th^ centile | 4/13 | 1/13 |
|  | 25^th^ centile | 4/13 | 1/13 |
|  | 75^th^ centile | 3/13 | 1/13 |
|  | 90^th^ centile | 3/13 | 0/13 |
|  | skew | 1/13 | 3/13 |
|  | kurtosis | 0/13 | 1/13 |
| D  /10^-3^mm^2^s^-1^ | median | 3/10 | 1/10 |
|  | mean | 3/10 | 1/10 |
|  | standard deviation | 1/10 | 0/10 |
|  | 10^th^ centile | 1/10 | 0/10 |
|  | 25^th^ centile | 2/10 | 1/10 |
|  | 75^th^ centile | 3/10 | 0/10 |
|  | 90^th^ centile | 4/10 | 0/10 |
|  | skew | 0/10 | 1/10 |
|  | kurtosis | 1/10 | 0/10 |
| *f* / % | median | 0/10 | 0/10 |
|  | mean | 0/10 | 1/10 |
|  | standard deviation | 1/10 | 0/10 |
|  | 10^th^ centile | 0/10 | 0/10 |
|  | 25^th^ centile | 0/10 | 1/10 |
|  | 75^th^ centile | 0/10 | 1/10 |
|  | 90^th^ centile | 0/10 | 1/10 |
|  | skew | 1/10 | 0/10 |
|  | kurtosis | 0/10 | 0/10 |
| D*  /10^-3^mm^2^s^-1^ | median | 1/10 | 0/10 |
|  | mean | 1/10 | 0/10 |
|  | standard deviation | 1/10 | 0/10 |
|  | 10^th^ centile | 0/10 | 0/10 |
|  | 25^th^ centile | 0/10 | 0/10 |
|  | 75^th^ centile | 1/10 | 0/10 |
|  | 90^th^ centile | 1/10 | 0/10 |
|  | skew | 0/10 | 1/10 |
|  | kurtosis | 0/10 | 2/10 |
| R_2_* / s^-1^ | median | 0/13 | 0/13 |
|  | mean | 1/13 | 0/13 |
|  | standard deviation | 0/13 | 0/13 |
|  | 10^th^ centile | 0/13 | 1/13 |
|  | 25^th^ centile | 0/13 | 1/13 |
|  | 75^th^ centile | 1/13 | 0/13 |
|  | 90^th^ centile | 1/13 | 0/13 |
|  | skew | 0/13 | 0/13 |
|  | kurtosis | 1/13 | 0/13 |
